# Therapeutic Effects of Cannabidiol on Methamphetamine Abuse: A Review of Preclinical Study

**DOI:** 10.22037/ijpr.2021.114918.15106

**Published:** 2021

**Authors:** Yasaman Razavi, Fariborz Keyhanfar, Ronak Shabani, Abbas Haghparast, Mehdi Mehdizadeh

**Affiliations:** a *Cellular and Molecular Research Center, Iran University of Medical Sciences, Tehran, Iran. *; b *Department of Pharmacology, Faculty of Medicine, Iran University of Medical Sciences, Tehran, Iran. *; c *Neuroscience Research Center, School of Medicine, Shahid Beheshti University of Medical Sciences, Tehran, Iran. *; d *Reproductive Sciences and Technology Research Center, Department of Anatomy, Iran University of Medical Sciences, Tehran, Iran. *

**Keywords:** Addiction, Cannabidiol, Methamphetamine, Therapeutic potential, Animal study.

## Abstract

As a strong and addictive psychostimulant, methamphetamine (METH) is often misused worldwide. Although relapse is the greatest challenge to the effective treatment of drug dependency, now, for METH addiction, there is not available accepted pharmacotherapy. To characterize a probable new target in this indication, a biological system comprised of endocannabinoids, known as the endocannabinoid system (ECS), has been advised. As a non-psychotomimetic Phytocannabinoid in *Cannabis sativa*, cannabidiol (CBD) has been used in preclinical and clinical studies for treating neuropsychiatric disorders. In this review article, we focus on the effects of CBD in the treatment of addiction in a preclinical investigation concerning the pharmaceutic effectiveness and the underlying mechanisms of action on drug abuse specially METH. Growing evidence shows that CBD is a potential therapeutic agent in reducing drug reward, as evaluated in conditioned place preference (CPP), brain-stimulation reward paradigms, and self- administration. Furthermore, CBD plays an effective role in decreasing relapse in animal research. Through multiple-mechanisms, there is a belief that CBD modulates brain dopamine responding to METH, resulting in a reduction of METH-seeking behaviors. As our studies indicate, CBD can decrease METH addiction-associated problems, for example, symptoms of withdrawal and craving. It is needed for conducting more preclinical investigations and upcoming clinical trials to entirely assess the CBD capability as interference for METH addiction.

## Introduction

While stimulants such as cocaine, amphetamine, and methamphetamine (METH) are some of the most used forbidden recreational drugs worldwide (1, 2), no established medication was found for using in treating stimulant use disorders as yet (3). Long-lasting METH abuse, even following the abstinence period, may bring about cognitive deficits (4, 5). Imaging studies of long-lasting abusers show decreases in the density of brain dopamine and dopamine transporter (4-6) and the density of serotonin transporter and decreases in brain serotonin (7). If there are opiates, although these treatment approaches, together with methadone, have bettered the outcome of drug dependence in opiate-dependent people, opiate craving in all patients cannot be successfully stopped through these treatment approaches. Considering this situation, new molecules need to be discovered for treating disorders of drug abuse without any successful treatment approach. 

During the past years, the enhanced identification of neural mechanisms involved in addictive disorders has been made available by the introduced modern knowledge and tools. The glutamatergic and dopaminergic systems are highly involved in the reinforcing outcomes of drugs and lengthy risk of relapse. Furthermore, the endocannabinoid system (ECBS) has an effect on the attainment and preservation of drug-seeking behaviors due to its function in reward and brain plasticity. According to the preliminary evidence, cannabidiol (CBD) can have healing outcomes for treating disorders of drug abuse (7-9). This review is intended to refine our present understanding of how CBD affects drug abuse such as METH and to discuss the existing data pointing to the possible effectiveness of CBD-based treatments for addiction treatment.

**Cannabidiol pharmacology**

 CBD is considered a non-intoxicating constituent in the company of more than 80 different cannabinoids that are present only in the cannabis plant (8, 9). THC is the cannabinoid most closely associated with euphoria, dependence, and mental health side effects associated with cannabis consumption (10-12). CBD, which is another main cannabinoid in the Cannabis plant, did not have THC psychoactivity (10, 13). CBD holds a complicated pharmacodynamic profile, as a whole, as we know to interact with an extensive type of molecular targets. Although no full investigation has been done on all these targets, it is widely believed that almost all of them (49%) are enzymatic, 20% are the membrane and cellular transporters, 15% are receptors, and 15% are ion channels (15). 

According to the reports of human studies, CBD has sedative, anxiolytic, antidepressant, mood stabilizer, and anti-craving properties (16-19). Lately, due to the anti-inflammatory and antioxidative (20) neuroprotective (21) properties of CBD and its hindrance to the rewarding effects of morphine (8), researchers have paid attention to it. The CBD effect of drug dependency has been explored by some studies. For example, the attenuated motivation of CBD to self-administration and relapse to METH (14). According to *Trigo et al. *’s study, CBD prevents the potential relapse in cannabis dependency (15). Furthermore, the potential of CBD in decreasing susceptibility to drug addiction and relapse has been shown (16). In *Razavi et al. *’s study, chronic ICV administration of CBD on impairments generated by METH in cognitive functions and recognition memory in mice chronically exposed to METH for the period of the abstinence (17) discovered the ability of CBD treatment for restoring spatial memory deficits. As these data denote, the rewarding effect and memory impairment of drug abuse is lessened by CBD treatment, suggesting a probable potential for combating relapse to drug-seeking. 

**Effects of Cannabidiol on reward circuitry**

According to some data, numerous neuronal circuits concerned with drug addiction are modulated by CBD. CBD blocks the brain’s reward system. Recent evidence demonstrated that high doses of CBD (10 and 20 mg/kg) significantly increased Intracranial Self-Stimulation (ICSS) threshold frequency in the medial forebrain bundle (9). This possibly will denote the anti-reward effect of CBD; nevertheless, in the mentioned study, a 5 mg/kg dosage CBD, although efficient to reduce the morphine effects, did not adjust the acute strengthening properties of cocaine (8); it is also consistent with a previous report suggesting that CBD does not induce conditioned place preference (CPP) and therefore lacks hedonic properties (18). Several animal works have studied that single or repeated CBD administration can decrease the rewarding effects of cocaine and METH. CBD can decrease drug intake and weaken relapse to drug-seeking behavior (14, 19-21). 

Furthermore, as a result of a rise in the expression level of hippocampal CB1Rs and *BDNF*, the repeated CBD administration not only decreases cocaine intake but also results in lasting neuroplasticity of the mesolimbic system (27). Unlike THC, CBD does not produce psychotomimetic properties and abuse potential (29), making it a promising candidate for future clinical use. Altogether, the CBD capability for reversing the raised movement of the mesolimbic DA reward system caused by the susceptibility to drug abuse can be a very important mechanism underlying its utility versus the addiction to psychostimulants and other drugs.

**Effect of Cannabidiol in addictions**

Drug addiction, known as mandatory drug-seeking, is a chronic condition that contains interchange drug withdrawal and relapse periods (30). Relatively few studies have examined the CBD effects on addiction and substance abuse in animals. CBD was examined for its anti-addictive properties in several animal models of cannabis, psychostimulants, opioid, alcohol, and nicotine addictions. Several studies have evaluated the CBD effect on drug dependency. For instance, previous studies have found the potentially relapse‐preventing effects of *Sativex* (THC/CBD) in cannabis dependency (23). In addition, CBD inhibits the reinstatement of cocaine and prevents the reinstatement of METH-induced CPP in rats (31).A recent study demonstrated that CBD treatment could prevent the reinstatement of Methylphenidate-induced CPP and produced shorter extinction latencies (22) . In an experiment with cocaine, the effects of a 10 mg/kg dose of CBD were evaluated on acquisition, consolidation, reconsolidation, extinction, and drug-primed reinstatement of cocaine using the drug CPP model, and the impact of CBD was examined in adult male mice. The results showed that CBD decreased preference of cocaine 20 days next treatment interruption, although there was no CBD effect on extinction, reconsolidation, or reinstatement of cocaine memory. These findings indicate that an acute 10 mg/kg dose of CBD has specific effects on cocaine memory processes. 

Moreover, it has been suggested that CBD can be utilized as an efficient and innovative therapy for destabilizing the memories connected with drugs triggering abuse, thus lessening the drug relapse risk (32). Some studies were indicated evaluating the CBD effects on alcohol drinking associated with relapse and addiction (28, 33), for example, the CBD effects on motivation for drinking alcohol (28) using the alcohol self-administration paradigm (34). These findings indicate that the strengthening properties, motivation, and relapse for the consumption of ethanol were diminished by the CBD administration, suggesting the ability of CBD for treating disorders of alcohol use. While significant preclinical data on opioid drugs and CBD in the animal are accruing, the consistent findings of opioid abuse show that that CBD diminishes symptoms of morphine withdrawal (35, 36), and even in combination with THC, CBD can reduce the abstinence scores even higher than THC alone (35, 36). In addition, acute CBD lessened cue-caused reinstatement of heroin seeking examined one day following injection (26). These studies prove that CPP is not promoted by CBD (37, 38), or the reinforcing efficacy of brain stimulation is not increased (8), which are both definitive characteristics of addictive substances. CBD interrelates with neurotransmitter systems, which are essential for the effects of opioids and psychostimulants. For instance, CBD can allosterically regulate δ and μ opioid receptors (23) and cannot inhibit uptake in striatal dopamine synapses (24). Previous research revealed a reversed decline in expressing intra-accumbal AMPA glutamate receptor GluA1 subunits in heroin-trained mice after treating with CBD (19). According to the extraordinary speculation, the augmented cocaine-seeking for the abstinence period is partly reliant on the moderately augmented expression of GluA1 subunits in the nucleus accumbens (NAc) (19, 5). Thus, CBD may have high potential as an adjunct to cue exposure therapies for disorders, such as addiction. 

**Effect of Cannabidiol in METH abuse**

For the implication of CBD on each phase of psychostimulant addiction, there are a few animal studies. The expansion of pharmacotherapies for treating stimulant use disorder has been in precedence in the studies conducted on addiction for more than 20 years, but the Food and Drug Administration (FDA) in the United States of America, or similar organizations in other countries did not have approved medication for this disease yet (42). The increased dopamine levels of the brain are the main mechanism of action related to the euphoria and abuse potential of METH; besides, as it can be suggested by a rising preclinical and clinical literature, blockers of dopamine uptake and releasers (methylphenidate, d-amphetamine, bupropion, modafinil, and methamphetamine) can be successful in the treatment of stimulant abuse and dependence (3). On the other hand, there is significant abuse and diversion potential for methylphenidate and d-amphetamine, and it seems that bupropion and modafinil have restricted clinical efficacy (3, 26). Consequently, to assist people with challenging stimulant use who are seeking treatment, we need new approaches. CBD inhibits the dopamine uptake, but this effect’s pharmacological relevance in a human being is unidentified (27, 28). CBD was not inherently hedonistic (18). Recently the 80 mg/kg CBD effects on reducing the motivation to self-administer METH and diminished methamphetamine-primed relapse to methamphetamine-seeking behavior following extinction were investigated in an animal study (14). Alternatively, according to *Parker et al. *’s study, THC and CBD potentiate the extinction of CPP learning provoked by amphetamine in the rats (18), and a CB1 receptor antagonist did not reverse this effect, which implies that additional neurochemical mechanisms possibly will be involved. Furthermore, as *Karimi* *et al. *’s study showed, the METH-induced reinstatement in extinguished rats could be suppressed by the ICV administration of the 10 μg/5 μL CBD through alteration of gene expression of cytokines, including *interleukin-1β, -6, -10* and *TNF-α* (29, 30). These cytokines are recognized to regulate the neuronal activities of monoamine neurons which include DA neurons in a straight line by using activating cytokine receptors placed on DA and other monoamine neurons and not directly through the release of neuroactive molecules from glia cells (31, 32). Therefore, it was pointed out that METH re-exposure increases the expression of proinflammatory cytokines such as *IL-1β* and *TNF-α*, which leads to the release of neurotransmitters concerned with the METH reinstatement. It turns out that CBD worsens this type of METH-provoked neuroplasticity in the mesocorticolimbic system of DA.

Mechanisms of specific receptors underlying CBD’s activity versus METH addiction-associated behaviors remain unreported yet. Data have exposed that the CBD administration into NAc can prevent METH-stimulated behavioral sensitization and hyperlocomotion and by regulation of downstream phosphorylation of the mTOR/p70S6 kinase signaling pathway inside the NAc shell (33). Moreover, animals conditioned with METH demonstrate CPP related to upregulation of the Sigma1 receptor and several intracellular molecules, e.g., CREB and p-CREB, p-GSK-3β AKT*, *p-AKT, and GSK-3β in the hippocampus, ventral tegmental area (VTA), prefrontal cortex (PFC), and the NAc. CBD inhibits CPP induced by METH in a fashion dependent on the dose. The expression levels of Sigma1R, p-AKT, p-GSK3β, and p-CREB were enhanced significantly in the CPP stimulated by METH (34). These results propose that CBD can opposite some of the METH-brought neuroplastic changes and may have therapeutic potential on METH-driven behaviors. In general, CBD studies possibly will suggest too much effect in addictive behaviors of psychostimulants in the relapse phase and seems not to be found on rewarding effects. According to a study recently conducted, the hyperactivity and behavioral sensitization induced by amphetamine were reversed by administering a dosage of 100 ng/0.5 μL CBD into the NAc (33). Undeniably, for opposing METH-seeking and craving, CBD not only can be a valuable agent but its antipsychotic efficacy (35), taking into consideration that psychosis is a pervasive challenge in heavy METH addicts, make an additional valuable feature available. Table 1 shows a summary of preclinical researches on the CBD effects in animals that were exposed to METH (36-38).

**Cannabidiol potential mechanisms of action**

**Figure 1:**
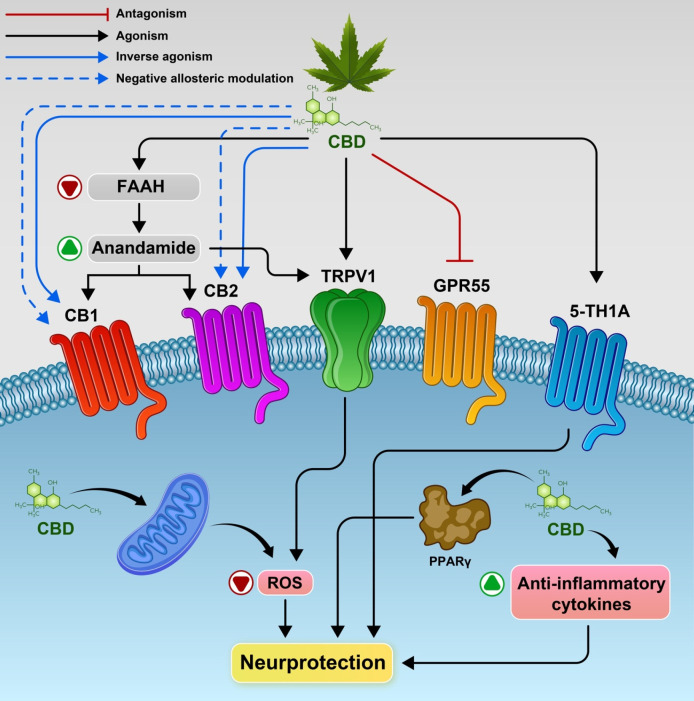
Potential mechanisms underlying CBD’s action and the main molecular targets. CBD inhibit, the enzyme which metabolizes anandamide *i.e.* FAAH. and activate CB1 and/or CB2 receptors indirectly. Also CBD may act as a CB1R negative allosteric modulator, a CB2R partial agonist or antagonist/inverse agonist. CBD activates the transient receptor potential channels (TRPV1), 5‐HT1A receptor and as antagonist of the receptor GPR55.Also promote PPARγ receptors, increased anti-inflammatory cytokines responses resulting in neuroprotection

While many possible mechanisms have been proposed, the CBD action mode is not completely comprehended yet. Similar to other cannabinoids, bell-shaped dose-response curves are produced by CBD, and diverse mechanisms can act as a result of its concentration or the concurrent existence of other cannabinoid ligands. The action of serotonin 5HT1A receptor, peroxisome proliferator-activated receptor-gamma *(*PPARγ*),* the metabotropic CB1 and CB2 receptors, and members of the TRPV family can be directly or indirectly regulated by CBD (39, 40). As indicated by recent evidence, withdrawal signs of METH-dependence may be reduced by acute administration of CBD, but the treatment must be elongated over time to facilitate METH quitting. Mostly hypothetically, the CBD ability in treating addictive disorders has been associated with modulating endocannabinoid, serotoninergic and glutamatergic systems (Figure 1).

**Cannabinoid receptors**

CBD includes a minimal affinity for CB1 and CB2, which are two recognized cannabinoid receptors. The ability of the CB1 agonists (WIN55212 and CP55940) to affect contractions at doses that were noticeably less than those of CBD required for activating cannabinoid receptors was attenuated by CBD (41, 42). As reported, CBD plays a role as an antagonist of CB1R agonists, for example, WIN-55212 and CP-55940 (43). Also, as CB1R internalization was inhibited by CBD (44), there is a hypothesis that the seen antagonistic activity may perhaps be based on negative allosteric modulation of CB1R instead of on orthosteric binding. According to the evidence obtained by these findings, *in-vitro* CBD acts as a non-competitive negative allosteric modulator of CB1R (41). More recently, a study reported that these actions are cannabinoid-receptor-mediated. Probably, CBD inhibiting fatty acid amide hydrolase (FAAH) activity increases the level of arachidonoylethanolamide (AEA), which actives CB1R (45). Moreover, CBD was determined to operate as a CB2R antagonist or inverse agonist (43). CBD includes a high-potency antagonist of cannabinoid-receptor agonists in the brain of mice and also in membranes from cells transfected with human CB2. Also, an inverse agonism is exhibited by CBD at the human CB2 receptor. Lots of the effects recorded with CBD, such as its anti-inflammatory properties, may be rationalized by these unexpected observations. Also, these findings report that CB2R antagonism attenuates CBD-produced neuroprotection (46).

**The 5-HT1a Receptor**

 CBD has been recently found that activating post-synaptic 5-HT1A receptors will possibly apply anxiolytic effects in the crosstalk between cannabinoids and serotoninergic signaling (34). The CBD’s anxiolytic properties have been proven in different animal models, such as the elevated plus-maze and conditioned emotional response (32, 33).

The 5-HT1A receptors play essential parts in the pathophysiology of depression, anxiety, and aggression. The agonist [3H] 8-OHDPAT from the cloned human 5-HT1a receptor is dislocated by CBD in a concentration-dependent manner. Contrastingly, agonist from the receptor in the same micromolar concentration range is not displaced by the major psychoactive component of cannabis, THC. CBD is considered as a modest-affinity agonist at the human 5-HT1a receptor; on the other hand, CBD increases the agonist serotonin, as well as GTPgS binding in this G-protein-coupled receptor (GPCR) system. Furthermore, the cAMP concentration at similar apparent levels of receptor occupancy is decreased by both CBD and 5-HT in this GPCR system negatively coupled to cAMP production (47, 48). Besides, the anti-craving effect of CBD may be also contributed by the agonist activity of CBD in the direction of 5HT1A receptors; similarly, the substance abuse relapses by regulation of the stress management, anxiety symptoms, and drug reward system are reduced by the agonist action of CBD in the direction of 5HT1A receptors (16). Eventually, the glutamatergic signaling by modulating endocannabinoid and serotoninergic systems might be regulated by CBD, and since dysregulating the glutamatergic transmission has been broadly associated with both abuse relapses and drug-seeking behaviors (16), this mechanism may be also involved in treating addictive behaviors (49). In rats, anti- aversive outcomes in the raised plus-maze and flight-induced by local electric stimulation are created by CBD administration into the dorsal parts of periaqueductal gray matter (dPAG). WAY-100635, a 5HT1A antagonist prevented these effects (50){Campos, 2008, Involvement of 5HT1A receptors in the anxiolytic-like effects of cannabidiol injected into the dorsolateral periaqueductal gray of rats}. Also, it seems that the basal ganglia (51), the bed nucleus of stria terminallis (52), the prelimbic PFC (53), and the dorsal raphe nucleus (9, 47), which are other brain regions, mediate the effects of CBD through 5HT1A receptors. Not long ago, *Magen* *et al. *verified that activating 5-HT1A receptors positioned in forebrain regions that include the hippocampus, and also, recovered cognitive and locomotor function weakened by bile-duct ligation was induced by CBD (5 mg/kg, i.p) (54). As a whole, these data denote that CBD possibly activates the 5-HT1A receptor, resulting in improving cognitive and functional impairment. 

**A Potent Antioxidant**

The well-known antioxidants are phenols, including resorcinols. Similarly, monophenols, plant cannabinoids, monophenolic ethers (like THC), or resorcinols (as CBD) are strong antioxidants. In a study, *Hampson* *et al. *(64) observed that CBD is a non-psychoactive ingredient of marijuana; also, it has a stronger effect than either α-tocopherol, which contains vitamin E, and is a dietary antioxidant, or ascorbate, which contains vitamin C, could prevent ROS-caused cell death and glutamate neurotoxicity. In a newer study (15), *Hamelink* *et al. *discovered that CBD safeguarded rats as opposed to hippocampal- entorhinal-cortical neurodegeneration while they were administered simultaneously with ethanol exposure. As they also have shown, this safeguard was not a result of NMDA-receptor antagonism, in the same way as other antioxidant NMDA antagonists, did not stop cell death and attached the CBD activity to its antioxidative impacts. By the original 1,1-diphenyl-2-picrylhydrazyl (DPPH) radical method explained by *Brand-Williams* *et al.*, CBD and THC were assessed in another study for antioxidant activity (57). Also, according to another study, CBD revealed stronger antioxidative ability than THC (55). These data indicate that CBD can be a possibly useful therapeutic agent for treating oxidative neurological conditions**.**

**PPARγ**

It was pointed out that CBD binds to the peroxisome proliferator-activated receptor-gamma (PPARγ) as it is supposed that the glitazone receptor is responsible for lipid storage and glucose metabolism (56, 57). PPARγ not only regulates inflammatory responses but also regulates the expression of genes associated with lipid and glucose homeostasis. Consequently, detected disorders of glucose metabolism and immune/inflammatory processes by PPARγ activation may be ameliorated by CBD (58), and it is supposed that some anticancer effects of CBD are mediated through interaction with PPARγ. CBD has also inhibited tumor cell viability.

**TRPV1 and the Effects of Cannabidiol**

Also, at least in some cases, CBD and other non-psychotomimetic phytocannabinoids are able to act through the transient receptor potential vanilloid (TRPV), which is a member of the ion channel receptor family. TRPV1 in vitro is activated and desensitized by CBD and cannabidivarin (CBDV) (59). Activation of TRPV1 receptors plays a role in the bell-formed dose-response curve of the anxiolytic activity of CBD. Treatment of animals with a TRPV1 antagonist prevented the absence of effects seen at elevated dosages of CBD (60). Also, it seems that TRPV1 plays a part in the anti hyperalgesic effects of CBD (61) besides in CBD effects on the disruption of sensorimotor gating aroused from NMDA antagonists (62). Evidence indicated that TRPV1 channels are on striatal GABAergic neurons and neurons of glutamatergic in the frontal cortex(63). It is suggested that CBD via TRPV1 may modify GABA release and brain glutamate, which leads to decreased cocaine reward. (64). Previous research showed that pretreatment with an antagonist of TRPV1(capsazepine) was in a position to stop CBD- induced reduction in the cocaine self-administration (65), proposing that activation of TRPV1 might as well come to the aid of the therapeutic effects from CBD.

**Neuroprotective effects as a selective therapeutic by Cannabidiol**

An essential system of action of neuropsychiatric medications to maintain the functions and structure of neural cells is constituted by neuroprotection, which promotes protection against protein aggregation, excitotoxicity, organelles damage, inflammation, and oxidative stress (66). It was proposed that by some intrinsic pharmacological properties, CBD impedes the THC effects. When CBD is administered alone, its hypnotic, anticonvulsive, neuroprotective, and hormonal effects, *i.e*., the increased corticosterone and cortisol levels, are produced spontaneously and support the hypothesis that CBD might have anxiolytic and/or antipsychotic effects (77). However, as *Niesink* and *van Laar* (10) verified, CBD did not have more or less effective in normal physiological processes. In order to be able to express the effect of CBD, the natural “tone” of the endocannabinoid system should be disrupted by a stimulus such as pain or a shock reaction, or another cannabinoid such as THC (67). 

An early study linking CBD to neuroprotection shows that CBD plays an antioxidant role against toxicity and/or oxidative stress generated by amphetamine (68, 69). The neuroprotective effects of CBD may perhaps also consist of neuroinflammatory mechanisms. Ab-induced neuroinflammation is diminished by CBD; also, hippocampal neurogenesis is promoted by CBD. It seems that these effects occur partially by activating receptors of PPARγ (70). Furthermore, neuroprotective effects in the cerebral malaria model are exhibited by CBD. Cognitive function rescue is promoted by CBD treatment with an increase in the expression of BDNF and decreased proinflammatory cytokines (TNF-α) and (IL-6) levels (71). In animal encephalopathy models, motor activity restored levels of 5-HT and BDNF, and cognition were improved by CBD by activating the 5HT1A receptor (72). While studies linking CBD to autophagy in neuropsychiatric conditions in quantity are insufficient, this process can be modulated by CBD (73, 74). Anticonvulsant effects associated with the activation of the hippocampal autophagy path in the chronic stage of pilocarpine-stimulated seizure were produced by CBD explicitly in the brain (75). 

**Table 1:** Effects of CBD in animals exposed to METH

**Evaluation method**	**dose of CBD**	**Main results**	**References**
CPP	5 mg/kg. i.p.	CBD potentiates the extinction of Amphetamine-induced CPP and this effect is not reversed by CB1 receptor antagonist	Parker *et al. * 2004
Psychomotor sensitization	100 ng/0.50 μL	CBD attenuates Amphetamine-induced sensitization in nucleus accumbens shell. CBD controls downstream phosphorylation of the mTOR/p70S6 kinase signaling pathways directly within the shell of NAc.	*Renard et al. *2016
Self-administration	20, 40, and 80; i.p. mg/kg	CBD decreases the motivation to self-administer METH and reduces METH-primed relapse after extinction.	*Hay et al. *2018
CPP	10 μg/5 μL	ICV microinjection of CBD supress the METH-induced reinstatement even in REM sleep deprived rats.	*Karimi et al. *2018
CPP	10 μg/5 μL	CBD treatment reduced the mRNA expression of cytokines in the PFC and HIP. Also, CBD treatment before REM sleep deprivation augments the *TNF‐α*, *IL‐1β*, *IL‐6*, and *IL‐10* levels in the HIP but diminishes *IL‐10* in the PFC.	*Karimi et al. *2020
CPP	10, 20, 40, and 80; mg/kg i.p.	CBD prevent METH-induced CPP and causes differential inhibitory responses in the cellular protein abundance of, p-AKT, Sigma1R, p-GSK3β, and p-CREB across various brain regions.	*Yang et al. *2020
Chronic exposure	10 and 50 μg/5 μL	ICV microinjection of CBD improves spatial memory and reverses short- and long-term memory that are impaired by chronic exposure of METH during abstinence	*Razavi et al. *2020
Chronic exposure	10 and 50 μg/5 μL	ICV administration of CBD enhance the mRNA expression levels of *BDNF/TrkB*; *RAF1*, and *NGF/TrkA* in the HIP during abstinence.	*Razavi et al. *2021
CPP	10, 50, 100, and 200 μg	ICV administration of CBD shifted the establishment of METH-induced CPP toward a lower dose. Concurrent CBD and METH treatments during sensitization phase established METH-induced CPP with sub-threshold doses of METH.	*Khaneghini et al. *2021
CPP	10 and 50 µg	Intra-CA1 microinjection of SCH23390 impairs CBD’s suppressive impact on both acquisition and expression phases of METH-induced CPP	*Anooshe et al. *2021
CPP	10 and 50 µg	CBD reduce METH-induced CPP. Intra-CA1 microinjection of sulpiride reversed the decreasing effects of CBD on METH-induced CPP in both acquisition and expression phases but more prominent in the expression phase	*Hassanlou et al. *2021

## Conclusion

To sum up, currently, we have inadequate evidence of the CBD’s possible therapeutic advantages of METH abuse and its frequently associated adverse symptoms. The quantity smallness of human research and the absence of clinical trials caused an apparent literature inadequacy. In preclinical research, CBD indicates pharmacological effectiveness in lessening propensity to relapse and drug reward. As reviewed, the action mechanisms of CBD are highly intricate and concerned with multiple receptors. Pharmacological (*in-vivo*) studies display that TRPV1, 5-HT1A, CB1, and CB2 receptors are essentially concerned with the mechanism of actions of the CBD. The mentioned receptors may modulate the activity of DA neurons in the DA and VTA release directly or in the NAc indirectly. Therefore, the mesolimbic DA system was able to operate as an ultimate target of anti-addiction effects of underlying CBD. There is an obvious necessity for other preclinical studies and forthcoming clinical trials to completely assess the CBD ability as an intervention therapy for METH addictive conditions. As a potential agent for the treatment of human addictive behaviors, evaluation of CBD should be more carefully carried out. They also confirm its low addictive risk as a new intervention for addiction as well as data that indicate CBD is not supporting on its own. For translating research findings into clinical settings, validating the CBD efficacy and safety in lessening the craving and relapse will be necessary for clinical and preclinical trials.

## Conflict of interest

The authors declare that there is no conflict of interest.

## Funding

We declare that there was no source of funding.
